# Supplementary material for: Management and Outcomes of Pancreatic Cancer in French Real-World Clinical Practice
Source: Cancers (Basel). 2022 Mar 25;14(7):1675. doi: 10.3390/cancers14071675 (PMC8996902; doi:10.3390/cancers14071675)
Supplement: Supplementary file 1 [file cancers-14-01675-s001.zip › cancers-1629066-supplementary.pdf]

Table S1. Description of toxicity types and consequences among patients receiving chemotherapy (n = 528).

	**No Change in or Interruption of Chemotherapy Regimen (No Toxicity / Mild Toxicity)**				**Change in or Interruption of Chemotherapy Regimen (Severe Toxicity)**			
	**Gem Alone**	**Gem_Ox/ Gem_Abra alone**	**FFX**	**Gem Adjuvant**	**Gem Alone**	**Gem_Ox/ Gem_Abra Alone**	**FFX**	**Gem Adjuvant**
Total	68	52	162	75	24	26	107	12
No toxicity	50	39	100	56	0	0	0	0
N	0	1	15	4	0	2	19	0
H	6	5	10	4	3	2	16	3
D	3	1	12	4	2	0	5	2
G	5	2	5	6	13	16	29	5
N H	0	1	3	0	0	1	3	0
N D	0	0	4	0	0	1	5	0
N G	0	0	1	0	0	0	4	0
H D	0	0	3	0	1	1	2	1
H G	3	1	0	0	0	2	6	0
D G	1	1	6	1	3	0	8	1
N H D	0	0	0	0	0	0	1	0
N H G	0	1	0	0	0	1	1	0
N D G	0	0	1	0	0	0	3	0
H D G	0	0	1	0	2	0	5	0
N H D G	0	0	1	0	0	0	0	0

N: Neurological toxicity
H: Haematological toxicity
D: Digestive toxicity
G: General toxicity (asthenia, allergy, headache, renal failure)

Gem alone= gemcitabine +/- oral capecitabine without resection,
Gem_Ox/Gem_Abra alone = gemcitabine+oxaliplatin or gemcitabine+paclitaxel without surgical resection
FFX = leucovorine+5-fluorouracil+irinotecan+oxaliplatin (FOLFIRINOX) with or without surgical resection
Gem adjuvant = gemcitabine after surgical resection
